# Using transects to disentangle the environmental drivers of plant‐microbiome assembly

**DOI:** 10.1111/pce.14190

**Published:** 2021-10-03

**Authors:** Jana Mittelstrass, F. Gianluca Sperone, Matthew W. Horton

**Affiliations:** ^1^ Department of Plant and Microbial Biology University of Zurich Zurich Switzerland; ^2^ Department of Environmental Science and Geology Wayne State University Detroit Michigan USA

**Keywords:** environment‐by‐environment interactions, *Fragaria vesca*, phyllosphere, rhizosphere, soil microbiota

## Abstract

Environmental heterogeneity is a major driver of plant‐microbiome assembly, but the specific climate and soil conditions that are involved remain poorly understood. To better understand plant microbiome formation, we examined the bacteria and fungi that colonize wild strawberry (*Fragaria vesca*) plants in North American and European populations. Using transects as replicates, we found strong overlap among the environmental conditions that best predict the overall similarity and richness of the plant microbiome, including soil nutrients that replicate across continents. Temperature is also among the main predictors of diversity for both bacteria and fungi in both the leaf and, unexpectedly, the root microbiome. Our results indicate that a small number of environmental factors, and their interactions, consistently contribute to plant microbiome formation, which has implications for predicting the contributions of microbes to plant productivity in ever‐changing environments.

## Abstract 2

The plant microbiome is understood to be heavily shaped by the environment, but the specific environmental factors that are involved are poorly understood. It also remains unclear whether the factors that drive leaf and root microbiome variation are similar and if these factors have the same effects on bacterial and fungal community diversity. Characterizing the microbiome of wild strawberry plants distributed across large transects on different continents revealed that the environmental factors that shape plant‐microbial communities are similar across large geographic scales.

## 1 INTRODUCTION

Plant‐microbial communities play a part in host nutrient uptake (Castrillo et al., 2017; Hiruma et al., 2016), responses to abiotic stress such as drought (Xu et al., 2018) and interactions with pathogens (Durán et al., 2018; Mendes et al., 2011) and herbivores (Schardl, Leuchtmann, & Spiering, 2004). Understanding the factors that shape the plant microbiome and identifying beneficial microbes within these communities (Zhang et al., 2019) could be key to developing sustainable agriculture or protecting plant productivity in challenging environments.

The factors that shape leaf and root microbiota seem to differ (Bergelson, Mittelstrass, & Horton, 2019; Wagner et al., 2016) but both communities are influenced by the local environment (Thiergart et al., 2020; Vorholt, 2012), seasonal variability (Walters et al., 2018) and the order in which taxa colonize the microbiome (Carlström et al., 2019; Fukami et al., 2010; Werner & Kiers, 2015). The discovery that plant genetic differences also play a role (Kuske et al., 2002; Redford, Bowers, Knight, Linhart, & Fierer, 2010; Walters et al., 2018) has inspired interest in identifying the specific plant genes that are involved (Bergelson et al., 2019; Horton et al., 2014; Wallace, Kremling, Kovar, & Buckler, 2018). If the climate and soil nutrients that affect the plant microbiome were better understood, then important environmental measurements could be used as covariates to reduce experimental noise, which would increase the statistical power to map influential genes (Igl et al., 2010; Wang et al., 2016). Doing so would also provide insights into the loci and environmental conditions that underlie genotype‐by‐environment (G × E) interactions, which are believed to heavily shape the plant microbiome (Wagner et al., 2016; Walters et al., 2018).

The wild strawberry *F. vesca* is common in forests, meadows, on hillsides and alongside trails and roads (Liston, Cronn, & Ashman, 2014). With extensive environmental variability across its distribution, *F. vesca* is an ideal model for investigating the ecological and environmental factors that influence leaf‐ and root‐microbial communities. Notably, the dominant sub‐genome within cultivated strawberry, an octoploid (*F*. × *ananassa*), derives from the diploid *F. vesca* (Edger et al., 2019), which further adds to the value of *F. vesca* as an emerging model system.

Here, we compare the leaf‐ and root‐microbial communities of *F. vesca*, using samples collected from populations in North America and Europe. We also measured the climate characteristics and soil chemistry of each population, which enabled us to examine how the environment influences microbial assembly at both local and global scales. In contrast with our expectations, our results reveal that many of the environmental conditions that predict plant‐microbiome diversity are shared across large geographic distances.

## 2 MATERIALS AND METHODS

### 2.1 Sample collection and processing

We collected *F. vesca* plants across two transects in Europe and North America. All of the European samples were collected between 12 and 21 June, 2017; the US samples were collected between 1 and 6 August, 2017. The coordinates of each sample are listed in Data S1. Three soil samples and eight plants were collected at each site using sterile technique and then stored on dry ice. All of the soil samples were collected in plant‐free areas and represent the bulk or unplanted soil microbiome. The samples were transferred to the lab on dry ice, flash frozen in liquid nitrogen and stored at −80°C until further processing.

Prior to DNA extraction, the leaves and roots of each sample were separated and placed in separate 50 ml Falcon tubes. To characterize epiphytes, we modified an earlier approach (Bodenhausen, Horton, & Bergelson, 2013; Qvit‐Raz, Jurkevitch, & Belkin, 2008). Briefly, each sample was washed three times in 15 ml of potassium phosphate buffer (pH 8, 0.1 M, 4°C). That is, 15 ml of buffer was added to each Falcon tube, which was vortexed for 5 s. The samples were then transferred to Steriflip filter tubes (0.22 μm Millipore Express plus Membrane, Millipore Corporation, US), and then the wash through was discarded. After two additional washes, the filter membranes were removed to characterize the epiphytes. To examine endophytes, the washed samples were removed from the filter tubes and the leaves were lyophilized for ~12 hr. Once dry, the samples were ground to a fine powder in liquid nitrogen and 1% PVP using a sterile mortar and pestle.

### 2.2 DNA extraction

The filter membranes (discussed above) were used as input material to extract the leaf and root epiphytic fractions. For endophytes, ~200 mg of the lyophilized and mortar‐ground tissue powder was used. Each sample was placed in a 2 ml Eppendorf tube containing 6–8 sterile glass beads (1.7–2 mm). The incubation buffer from Qiagen DNA Isolation Kits (see below) was added to the tubes, and the mixture was homogenized twice for 30 s at 1400 rpm (Geno/Grinder 2010, SPEX SamplePrep). The samples were incubated at 65°C for 10 min to increase cell lysis, and, after cooling (4°C), the samples were homogenized once more with a MoBio vortex adaptor for 15 min at maximum speed. All of the soil samples were extracted using PowerSoil DNA Isolation kits (Qiagen). The plant samples were extracted using a combination of PowerSoil DNA Isolation kits and PowerPlant Pro‐htp 96 kits (Data S1) using the manufacturer's protocol. After homogenization, the samples processed using PowerPlant kits were loaded into the 96‐well plates before the first centrifugation step.

### 2.3 Amplicon sequencing

The extracted DNA was quantified using high sensitivity Qubit assays (Invitrogen) and amplified using a two‐step PCR protocol (Gohl et al., 2016). To characterize bacteria, we amplified the hypervariable regions V3 and V4 of the 16S ribosomal RNA gene using the primers V3F (Chakravorty, Helb, Burday, Connell, & Alland, 2007) and 799R (Chelius & Triplett, 2001). To identify fungi, the primers ITS1F (Gardes & Bruns, 1993) and ITS2 (White, Bruns, Lee, & Taylor, 1990) were used to sequence the first internal transcribed spacer (ITS1). The primers for the first PCR (PCR1) of the V34 region included *V3F_1*: [FOR]‐8N‐**AG**CCAGACTCCTACGGGAGGCAG and *799R_1*: [REV]‐8N‐**CG**CMGGGTATCTAAT‐CCKGTT. During PCR1 of ITS1, we used the primers *ITS1F_1*: [FOR]‐8N‐**GG**CTTGGTCATTTA‐GAGGAAGTAA and *ITS2_1*: [REV]‐8N‐**CA**GCTGCGTT‐CTTCATCGATGC. The sequences (5′ ‐ 3′) include a 2‐bp linker (shown in bold) at the 5′ end of each marker gene primer. The label [FOR] represents Illumina sequence: CTTTCCCTACACGACGCTCTTCCGATCT, while [REV] represents the Illumina sequence: GGAGTTCAGACGTGTGCTCTTCCGATCT. Exactly 8N refers to the eight degenerate bases between the Illumina adaptor and linker.

PCR1 was performed using an initial 5 min denaturation at 95°C, followed by 25 cycles of denaturing (98°C for 20 s), annealing (55°C for 30 s) and extension (72°C for 30 s). A final elongation step was performed for 7 min at 72°C before storing the samples at 4°C. The fragments were amplified with the KAPA HotStart PCR Kit (Roche), using 5 μl of 5x KAPA HiFi buffer, 0.75 μl of 10 mM dNTPs, 0.75 μl of each primer (both 10 μM), 0.5 U Hotstart Taq and 2 μl of template DNA. Two replicate PCRs were performed with each sample, each in a total volume of 25 μl. After PCR, the replicates were pooled, visualized on an agarose gel and cleaned using solid phase reverse immobilization (SPRI) magnetic beads (Deangelis, Wang, & Hawkins, 1995) at the Genetic Diversity Center (GDC) laboratories (ETH, Zurich, Switzerland).

During the second PCR (PCR2), 5 μl of cleaned PCR1 product was amplified using 25 μl of KAPA HotStart Ready Mix (Roche), 10 μl of PCR grade water, the forward primer (0.4 μM, 5ul) AATGATACGGCGACCACCGAGATCTACAC and the reverse primer (0.4 μM, 5ul) CAAGCAGAAGACGGCATACGAGAT; these primers were attached to barcodes from the Illumina TruSeq HT and Amplicon kits (D501‐D508, D701‐D712, A501‐A508, and A701‐A712; Data S1). The primer binding sites for PCR2 ([FOR] and [REV] discussed above) were located downstream of the barcoded indices. PCR2 was performed using an initial 5‐min denaturing step (98°C), followed by eight cycles of denaturing (98°C for 30 s), annealing (55°C for 30 s) and extension (72°C for 30 s). A final extension step was performed for 5 min (72°C). The final PCR products were cleaned with SPRI beads (as discussed above), and the libraries were quantified on a Spark microplate reader (Tecan Group Ltd, Switzerland) using the Qubit broad range assay. Then, the libraries were normalized and pooled on a BRAND (Wertheim, Germany) liquid handling station and visualized on an Agilent TapeStation. The final concentration of each library was adjusted to 4 nM before sequencing on an Illumina MiSeq instrument. In total, five 16S MiSeq libraries and four ITS1 MiSeq libraries (Data S1) were sequenced at the GDC and Functional Genomics Center Zurich (FGCZ) in Switzerland.

### 2.4 Sequence data processing

The demultiplexed sequences have been deposited in the European Nucleotide Archive (ENA) under accession code: *PRJEB39612*. QIIME2 (Bolyen et al., 2019) was used during data processing of the demultiplexed sequences (version qiime2‐2018.6). First, after importing the demultiplexed sequences of each run, the phylogenetic primers were removed using *cutadapt* (version 1.16 implemented in QIIME2). These trimmed sequences were then denoised and merged using DADA2 (Callahan et al., 2016). The sequences from each library, for each kingdom, were then truncated to 219 and 221 bp from the forward and reverse reads, respectively.

After merging the sequences within each run, we merged the results from each separate run for each separate kingdom. To identify bacteria, the bacterial sequences were classified using SILVA (Quast et al., 2013); the fungal sequences were classified using the UNITE (Nilsson et al., 2019) database (version November 18, 2018). The sequences were clustered into phylotypes that share 97% nucleotide similarity using *vsearch* (v2.7.0). We excluded sequences that were unassigned at the kingdom level, assigned to Chloroplast at the class level, or assigned to Mitochondria at the family level.

### 2.5 Environmental data

Data from the FAO GeoNetwork (Food and Agriculture Organization of the United Nations, 2012), the USGS (USGS digital elevation dataset SRTM mission, 2020), Worldclim (Fick & Hijmans, 2017) and other sources were used during all environmental analyses (Table S2 and Data S2). Data in raster format (average temperature, maximum temperature, minimum temperature, precipitation, solar radiation, water vapour pressure, wind speed) were first aggregated into monthly average datasets to create seasonal average variables. To estimate actual and potential seasonal evapotranspiration, the data, which are available as gridded point shapefiles (Global GIS: Global Climate Database: Actual evapotranspiration in EarthWorks, n.d.), were first transformed into continuous gridded surfaces using the ordinary kriging geostatistical interpolation method. Kriging assesses the uncertainty of the predictions at each location using a cross validation approach. The Geostatistical Analyst tools package available in ESRI ArcGIS Desktop 10.7.1 was used to model surfaces (ESRI, California, USA). Variables related to slope and aspect are based on data from NASA's shuttle radar topography mission (SRTM), using the 30 m elevation dataset. To facilitate interpretation, the variable aridity was converted into ^**−**^1 × aridity, and thus larger values are consistent with higher aridity. The cosine and sine of the slope's aspect were used to estimate the northing and easting of slope direction, respectively. The final 21 climatic variables were used to interpolate the conditions that each plant experienced based on its GPS coordinates, using the spatial overlay functions available in the Spatial Analyst package within ArcGIS.

Three soil samples were collected at each field site. One half of each sample was used to sequence soil microbial DNA (as discussed above). The remaining material was analysed by Labor für Boden and Umweltanalytik (LBU; Thun, Switzerland) to characterize the soil conditions at each site. Data S2 lists the raw data for the final 49 climate, geographic and soil variables used during analysis, while Table S2 contains the descriptions of each of the variables.

### 2.6 Microbiome structure

To identify taxonomic groups enriched in either the soil, root or leaf compartment (Data S1), we fit quasi‐Poisson generalized linear models using the function *glm* within *R* (R Development Core Team, 2012). To control for differences in sequencing effort among samples, we set the offset argument in *glm* to the log of the number of sequencing reads for each sample.

To perform center log ratio (CLR) normalization, we used the *aldex.clr* function available in the *R* package ALDEx2 (Fernandes et al., 2014). Principal component analyses (PCAs) were performed with the *R* function *prcomp* using CLR transformed data after removing singletons. Due to differences in the composition of the leaf, root and soil microbiome, the heatmaps (Figure 2b,d and Figure S4) focus on the most abundant 100 phylotypes in either the soil, root or leaf microbiome. The overlap among the top 100 taxa from these three separate compartments (i.e., taking the *union* of phylotype‐ids) results in 167 total bacterial and 214 total fungal phylotypes.

For each microbial habitat, we examined the overall similarity (that is, beta diversity) of all samples using redundancy analysis (RDA) with the functions *rda* and *Condition*, both of which are available in the *R* package vegan (Oksanen et al., 2011). In *rda*, the function *Condition* was included on the right side of all formulae to control for the effects of latitude and longitude. This initial RDA model was then used as input in the function *ordiR2step*, also available in vegan, to perform forward selection (*n* = 99,999 permutations). To control the false discovery rate (FDR, *q* ≤ 10%) of forward selected variables, we implemented the Benjamini and Hochberg method (Benjamini & Hochberg, 1995).

### 2.7 The distribution of microbes

Before examining the relationship between CLR enrichment (a measure of abundance) and the pattern of sharing across transects (Figure S5), we corrected for differences in sequencing depth among samples. To do so, each sample was resampled once to contain 5,000 reads, using the relative abundance distribution in the raw data to model the probability of sampling each taxon. The top shared and unique taxa are illustrated in Figure S5. Figure 3 shows the pattern of sharing across transects using the raw data.

### 2.8 Analyses of alpha diversity

To investigate whether and how strongly environmental variability affects richness in the microbiome, we examined the number of epiphytic or endophytic taxa in the leaf and (separately) the root microbiome. These count data were investigated using quasi‐Poisson generalized linear models with the *R* function *glm*. To correct for differences in the number of reads among samples, the offset argument within *glm* was set to the log of the number of sequencing reads for each sample. To analyse the total number of bacteria and fungi, we included the factor kingdom before testing the effect of each environmental variable or any interactions between environmental variables.

The *mantel* function (*n* = 99,999 permutations) in vegan was used to perform Mantel tests on the −log_10_ (*p* value) matrices from environment × environment (E × E) interaction tests, after setting the diagonal of each matrix to 0.5. To identify the environmental variables that underlie the Mantel *r* result for the rhizosphere, we iteratively removed the row and column corresponding to each individual environmental variable from these matrices and then repeated the Mantel test for each missing variable.

## 3 RESULTS

To characterize the wild strawberry microbiome, we established two transects (Figure S1), consisting of 12 populations in North America and 14 populations in central Europe (Table S1). Three soil and eight whole‐plant samples were collected from each population, and the leaves and roots of each plant were then separated into their epiphytic and endophytic compartments using sterile techniques.

To classify bacteria and archaea, we amplified and sequenced the V3‐4 region of the 16S ribosomal gene using Illumina sequencing. Fungal community profiles were created by sequencing the first internal transcribed spacer (ITS1) within eukaryotes. A significant proportion of next‐generation sequencing reads are expected to contain errors due to intrinsic technical artefacts (for example, cross‐talk between clusters (Wang, Wan, Wang, & Li, 2017)) and run‐to‐run variability due to polymerase errors (Ma et al., 2019) or incomplete washes between cycles (Pfeiffer et al., 2018). Moreover, microbial genomes often contain multiple copies of genetically divergent ribosomal DNA (Schoch et al., 2012). We therefore clustered the sequences into species‐level phylotypes that share ≥97% pairwise nucleotide sequence similarity. After omitting singletons, we identified 7,000 bacterial and 13,162 fungal phylotypes across all samples.

### 3.1 The bacteria and fungi within the wild strawberry microbiome

**Figure 1:**
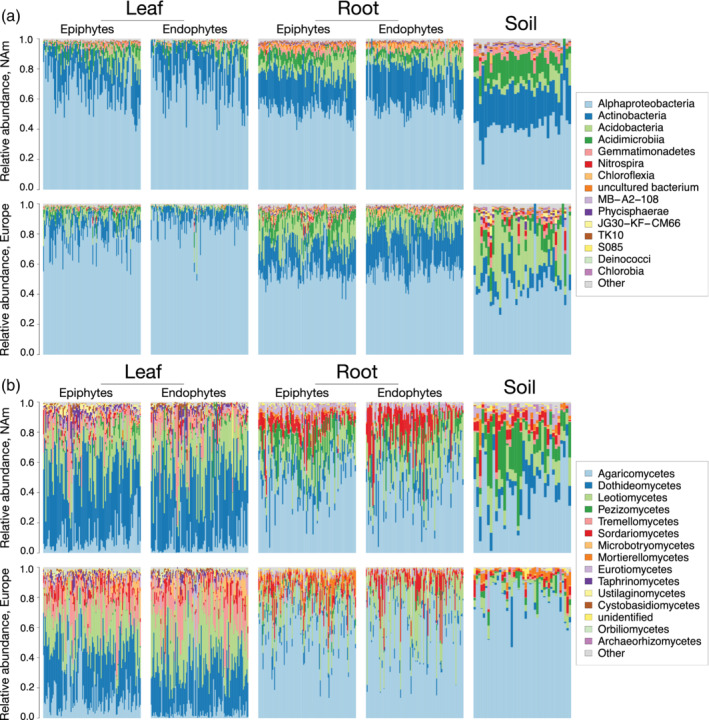
The taxa in the *F. vesca* microbiome. The relative abundances of (a) bacterial and (b) fungal classes in the leaf, root and soil microbiome. The North American (NAm) and European samples are plotted in the top and bottom row of each panel, respectively [Colour figure can be viewed at wileyonlinelibrary.com]

Although our main aim was to understand the environmental conditions that shape plant‐microbiota, we first used quasi‐Poisson generalized linear models to evaluate differences in the composition of the soil, root and leaf microbiome. We found broad differences between organs. For example, Proteobacteria are enriched in leaves compared with roots (for epiphytes, *p* = 2.3 × 10^−21^), while Actinobacteria (*p* = 5.45 × 10^−7^) and Acidobacteria (*p* = 9.32 × 10^−6^) are more common in roots (Figure S2 and Data S1). In particular, the *F. vesca* microbiome is heavily colonized by the class Alphaproteobacteria (Figure 1a), which is more prevalent in leaves than roots (*p* < 2.8 × 10^−22^).

In soil, the proportion of Alphaproteobacteria is similar across transects, but the abundances of Actinobacteria and Acidobacteria vary widely; specifically, the Actinobacteria are more abundant in the soils of North American sites (*p* = 4.05 × 10^−24^), while the Acidobacteria are more abundant in European sites (*p* = 3.14 × 10^−14^). Despite strong transect‐specific differences in soil communities, however, these taxa are found in increasingly consistent proportions in the root‐epiphytic (hereafter rhizosphere) and endophytic compartments of each transect (Figure S3 and Data S1), which demonstrates that assembly can be similar across large geographic distances. In contrast, compared with bacteria, the abundances of fungi in the rhizosphere and soil are roughly similar (Figures S2 and S3 and Data S1). Along each transect, the Agaricomycetes are more abundant in roots (Figure 1b), while the Dothideomycetes and Leotiomycetes are more abundant in leaves.

**Figure 2:**
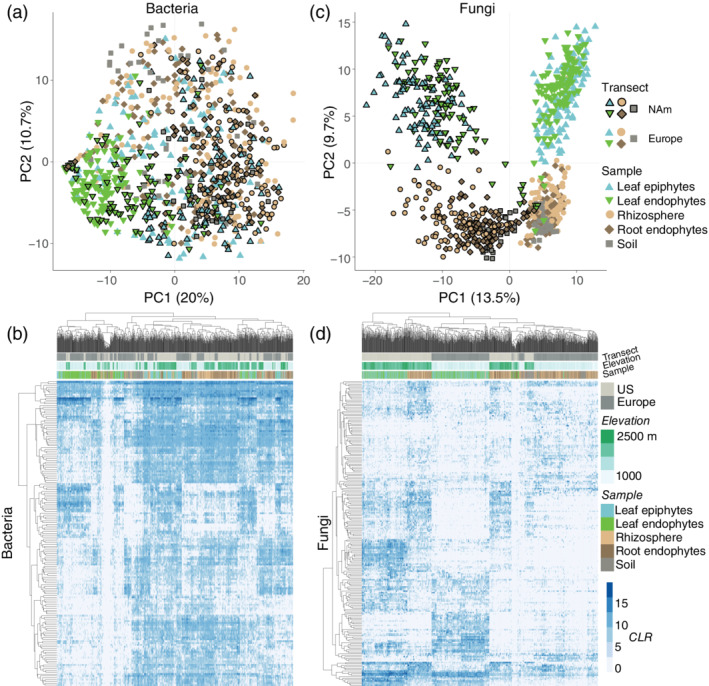
The structure of the wild strawberry microbiome. (a) PC1 and PC2 from PCA of the bacterial community (top *n* = 250 phylotypes), after center log ratio (CLR) transformation. (b) The distribution of the top bacteria within the soil, root and leaf microbiome. (c) PC1 and PC2 from PCA of the fungal community, as in panel a. (d) The distribution of the top fungi in the soil, root and leaf microbiome. The samples from North America are drawn with a black border in panels a and c [Colour figure can be viewed at wileyonlinelibrary.com]

PCA of the top (that is, the most heavily sequenced taxa) 250 bacteria revealed little differentiation among samples. Instead, leaf epiphytes overlap soil and root samples (Figure 2a), which suggests that root and soil bacteria also disperse to the leaf surface. Moreover, despite the physical distance between transects (~8,500 km), samples from both continents overlap, which indicates that wild strawberry is colonized by many of the same bacteria across its range. Indeed, many bacteria are found in the leaves and roots of both transects (Figure 2b, the labels are shown in Figure S4), although some strains are enriched in one organ or along one transect. As an example, a member of *Bradyrhizobium*, a common genus in soils (Delgado‐Baquerizo et al., 2018), is the dominant root bacterial phylotype along both transects. This bacterium is also found in the phyllosphere but at a lower median abundance than other bacteria. Likewise, a widespread *Sphingomonas* phylotype dominates the phyllosphere of both North American and European samples. Although this phylotype is found in roots, it usually occurs at a lower abundance than other taxa.

Compared with bacteria, PCA of fungal communities results in more clearly clustered samples (Figure 2c), as PC1 separates samples by transect and PC2 separates leaf and root samples. These patterns are driven by differences in the enrichment and presence–absence of taxa (Figure 2d). Notably, only two of the top 10 fungi in leaves are also among the top 10 root fungi, and only one of these is common along both transects, a member of the *Mycosphaerella*, a genus that includes the cultivated strawberry pathogen *M. fragariae* (Ehsani‐Moghaddam, Charles, Carisse, & Khanizadeh, 2006).

**Figure 3:**
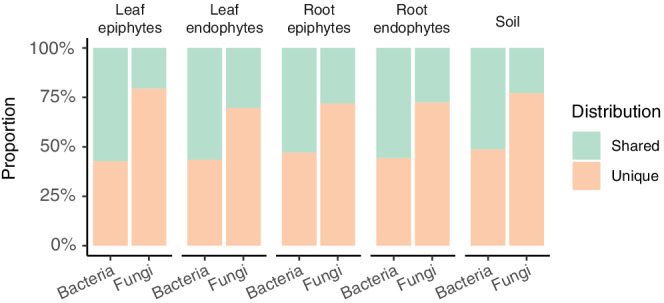
Differences in the distribution of bacteria and fungi. The proportion of bacteria and fungi that are shared or unique across transects, focusing on the top 250 taxa within each community [Colour figure can be viewed at wileyonlinelibrary.com]

Taken together, these patterns are consistent with the hypothesis that fungi are dispersal limited compared with prokaryotes (Coleman‐Derr et al., 2016). To explore this possibility further, we compared the distribution patterns of bacteria and fungi. Specifically, we examined whether the top 250 taxa from each kingdom were found along one or both transects. Overall, we found that the top bacteria are more widely distributed than the top fungi (Figure 3 and Figure S5), as 50% of the top bacteria, but fewer than 30% of the top fungi, were detected along both transects.

### 3.2 The drivers of plant microbiome variation

We next examined how soil, climate and geography influence the overall similarity (beta diversity) and the number of taxa (richness, a measure of alpha diversity) within the microbiome. The environmental measurements include the availability of soil nutrients, seasonal estimates of precipitation and temperature, slope and more (Figure S6, Table S2, and Data S2). Each compartment of the microbiome was analysed separately to investigate whether the factors that drive microbiome assembly differ between leaves and roots, epiphytes and endophytes, and bacteria and fungi. Using transects as replicates also enabled us to consider the local and global importance of each environmental factor.

**Figure 4:**
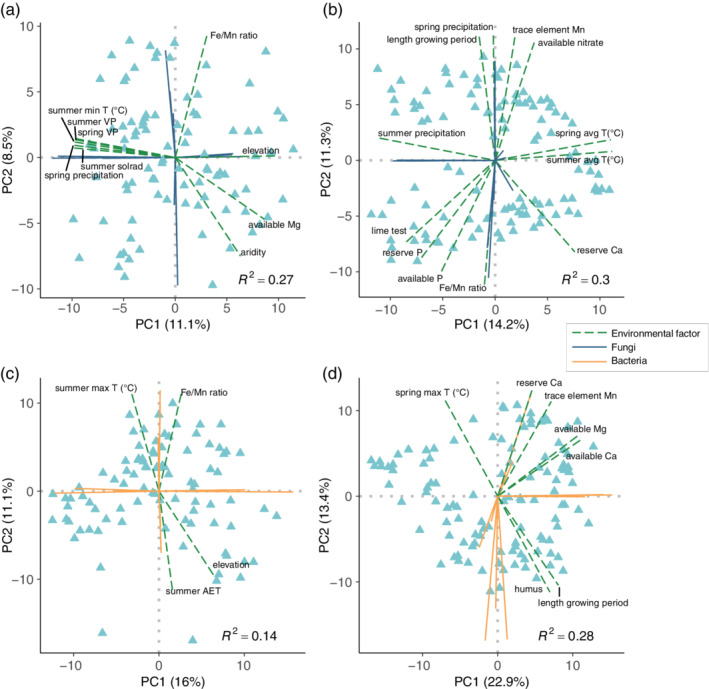
The environmental factors that predict the overall similarity of leaf epiphytes. The results from PCA of (a, b) fungi and (c, d) bacteria on the leaves of (a, c) North American and (b, d) European samples. Each PCA was performed with the top 250 taxa within each community. The green dashed lines indicate the environmental variables identified using a forward selection procedure (*n* = 99,999 permutations; FDR *q* ≤ 0.10) [Colour figure can be viewed at wileyonlinelibrary.com]

First, we performed PCA of the top 250 taxa within each microbial compartment. A forward selection procedure was used to understand which factors affect community similarity while adjusting for the geographic coordinates of each sample to control for dispersal limitation. With leaf‐epiphytic fungi (Figure 4ab), for example, we explained 27% of the variation in North American and 30% in European sites (FDR ≤ 10%). Although much of this variation is explained by local environmental variability, some environmental conditions surprisingly predict beta diversity along both transects (Figures S7 and S8). For example, in the case of leaf‐epiphytic fungi, variation along PC1 is consistently associated with temperature and precipitation, while variation along PC2 is linked to the ratio of iron to manganese (Fe/Mn) and other local factors. Similarly, temperature and manganese predict the structure of bacterial communities (Figure 4c,d and Figure S7). Manganese was recently found to promote carbon fixation in bacteria (Yu & Leadbetter, 2020), which may explain its role in plant‐microbiome assembly. Compared with epiphytes, analyses of endophytes generally resulted in weaker *R* ^2^ values, which implies that endophytes are more strongly influenced by unmeasured abiotic variables, the granularity of our measurements and/or host‐genetic effects. Nevertheless, we found a few consistent predictors of endophytic similarity across transects, such as wind speed for leaf bacteria and water vapour pressure for fungi (Figure S7).

**Figure 5:**
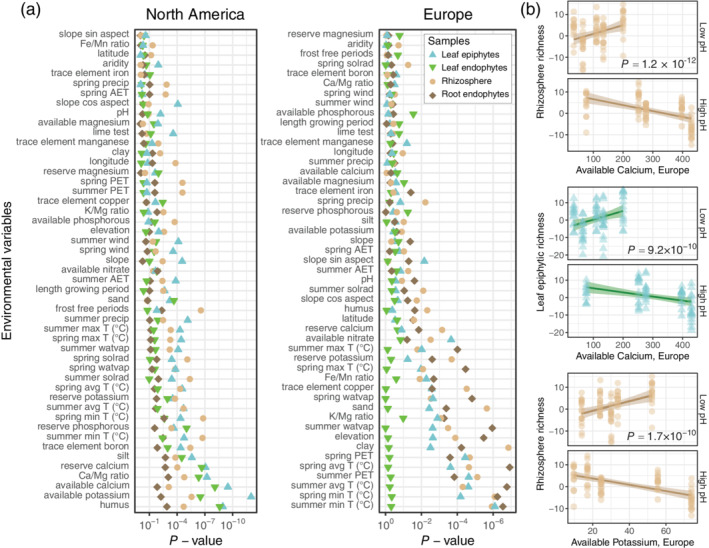
The relationship between the environment and plant‐microbiome richness. (a) The environmental variables that are most strongly associated with bacterial and fungal richness in the microbiome of North American and European samples. The variables are sorted by median *p* value. (b) Richness is likewise linked to interactions between environmental variables including Ca and K availability with pH. Each analysis was performed using quasi‐Poisson models that control for the number of reads per sample [Colour figure can be viewed at wileyonlinelibrary.com]

The number of microbes (that is, richness) within each microbiome compartment differs among habitats (Figure S9). In addition, richness is strongly predicted by the environment. We found that bacterial and fungal richness are predicted by many of the same environmental factors. For example, variation in both the bacterial and fungal communities is driven by the amount of humus, the Ca/Mg ratio and other factors in North America (Figure S10). In Europe, both bacterial and fungal richness are predicted by variables related to evapotranspiration, clay and copper. To improve power, we combined both kingdoms into one analysis, which revealed that the variables that best predict alpha diversity are related to temperature (Figure 5a), which is consistent with analyses of beta diversity (Figure 4, Figures S7 and S8). On average, richness in the microbiome is lower for plants collected from areas that have historically experienced colder temperatures.

Apart from temperature, leaf‐ and root‐microbiome richness appears to be driven by local environmental heterogeneity along each transect (Figure 5a). As one example, diversity in the leaf microbiome is associated with the availability of the cations Ca and K in North American populations; however, neither mineral has a strong main effect in European sites, where the abundance of each cation is highly variable (Figure S11). This raises the possibility that the effects of these elements are masked by other environmental conditions in our European sites.

We thus asked whether the soil and climatic drivers of microbiome assembly are context‐dependent. To do so, we used quasi‐Poisson generalized linear models to identify environment‐by‐environment (E × E) interactions associated with total richness. For example, despite no evidence of a relationship between richness and Ca availability along the European transect, a crossover interaction between soil pH and Ca predicts rhizosphere (*p* = 1.21 × 10^−12^) and leaf‐epiphytic (*p* = 9.23 × 10^−10^) richness (Figure 5b). Like Ca, a weak main effect of K availability in Europe is outweighed by crossover interactions within the rhizosphere between K and lime (*p* = 9 × 10^−13^), water vapour pressure (*p* = 9.1 × 10^−11^) and pH (Figure 5b; *p* = 1.7 × 10^−10^). Soil pH is less acidic and, like the availability of Ca and K, less variable along our North American transect (Figure S11a–c). Likewise, the abundance of copper predicts diversity in our European but not North American sites (Figure 5a). Nevertheless, copper is part of several E × E interactions linked to richness along the North American transect (Figure S11 and Table S3).

Overall, we found several E × E interactions where one of the two variables in the interaction independently predicts diversity along the other transect as a main effect (Figure 5, Figure S11, and Table S3). Moreover, the E × E interactions that predict rhizosphere diversity partly overlap across transects (Mantel *r* = 0.13, *p* = 0.01; Figure S11d,e). Taken together, this suggests that the environmental conditions that shape microbial communities are similar across large geographical scales. Iteratively dropping out environmental variables one at a time and repeating Mantel tests reveals that lime, which buffers soil pH, is the factor that is most responsible for the similarity of E × E interactions across continents (Figure S11f).

## 4 DISCUSSION

Given the impact of microbiota on plant health and productivity (Castrillo et al., 2017; Hiruma et al., 2016; Mendes et al., 2011; Xu et al., 2018), it is essential to discover the factors that contribute to plant‐microbiome assembly. To characterize the composition of wild strawberry's (*F. vesca*) leaf and root microbiome, we collected plants from undisturbed populations in North America and Europe. Our results demonstrate that plants collected from different continents host many of the same taxa in their leaf‐ and root‐microbial communities, although there is a stronger overlap among bacterial than fungal communities. These results, and similar patterns in the soil microbiome, suggest that fungi and bacteria are differentially affected by ecological processes such as dispersal limitation, demographic drift or environmental selection.

To better understand how the environment affects plant‐microbiome assembly, we characterized the climate and soil conditions in wild strawberry populations in North America and Europe. We then sought to understand the conditions that best predict the overall diversity of and within microbial communities. Our results indicate that temperature is a main driver of microbiome assembly, which has wide‐ranging implications for predicting the outcome of climate warming and reducing the effects of environmental change on plant productivity. Notably, temperature is a key predictor of the overall similarity (beta diversity) and the number of taxa (richness, a form of alpha diversity) within the bacterial and fungal communities of both leaves and roots.

Our study also demonstrates that many of the climate conditions, soil nutrients and E × E interactions linked to plant‐microbiome variation are shared across continents, which supports the feasibility of using microbiome management to promote plant productivity. As one example, we found a strong overlap in the climatic and soil factors that predict the similarity of fungal communities on the leaf surface. This overlap in predictors, combined with the observation that there is limited overlap among the fungal taxa that actually inhabit these communities, implies that the role of the environment in shaping microbial assembly is more general than is often assumed.

Genetic differences among plants also contribute to microbiome assembly (Balint‐Kurti, Simmons, Blum, Ballare, & Stapleton, 2010; Bergelson et al., 2019; Horton et al., 2014; Wagner et al., 2016; Wallace et al., 2018; Walters et al., 2018), but few of the genes involved (Zhang et al., 2019) have been identified or validated in natural settings. Although the effects of host genes may differ in magnitude in different environments, incorporating environmental covariates (for example, diet and smoking status) in analyses has been shown to improve power in studies of human disease (Igl et al., 2010) and the human microbiome (Wang et al., 2016). To accelerate the discovery of genes that influence the plant microbiome, future studies should similarly model important environmental conditions such as temperature and soil chemistry. Doing so will also enable the G × E interactions that are reported to influence microbiome assembly (Tabrett & Horton, 2020; Thiergart et al., 2020; Wang et al., 2016) to be better understood.

## CONFLICT OF INTEREST

The authors have no conflict of interest to declare.

## Supporting information


**Data S1.** Supporting information.

**Data S2.** Supporting information.

## Data Availability

The sequencing data have been deposited in the European Nucleotide Archive (ENA) under the accession id *PRJEB39612*. All of the other data needed to evaluate the conclusions in the paper are present in the paper and/or the Supplementary Information. The scripts used to examine the data are available at: https://bitbucket.org/horton_lab/microbiome_survey.git.
