# Supplementary material for: Using transects to disentangle the environmental drivers of plant‐microbiome assembly
Source: Plant Cell Environ. 2021 Oct 3;44(12):3745–55. doi: 10.1111/pce.14190 (PMC9292149; doi:10.1111/pce.14190)
Supplement: Supplementary file 2 — Data S2. Supporting information. [file PCE-44-3745-s002.docx]

**Using transects to disentangle the environmental drivers of plant microbiome assembly**

Jana Mittelstrass, F. Gianluca Sperone, and Matthew W. Horton^*^

^*^Correspondence should be addressed to M.W.H. (horton.matthew.w@gmail.com)

***Supporting Information***

**This file includes:**

Supplementary Figures 1 - 11

Supplementary Tables 1 - 3

The description of Supplemental dataset files 1 – 3

Supplementary References

**Supplementary Figure 1: The field sites established during the study.** **(a)** The North American transect contains 12 field sites. (b) The European transect contains 14 field sites.


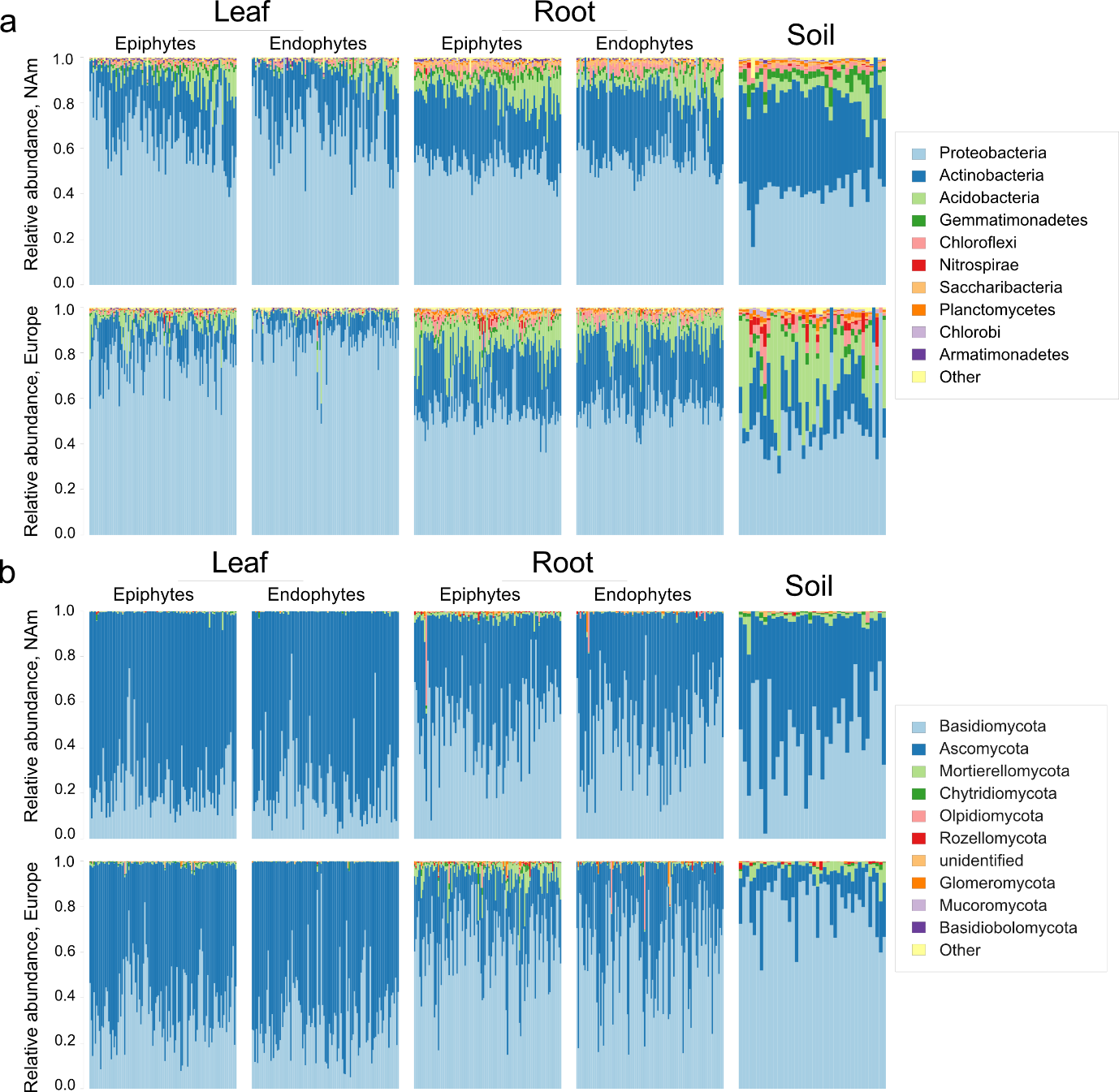


**Supplementary Figure 2: The phyla in the *F. vesca* microbiome.** The relative abundances of **(a)** bacterial and **(b)** fungal phyla in the leaf, root, and soil microbiome. The North American (NAm) samples are plotted in the top row of each panel, and the European samples are plotted in the bottom row.

**Supplementary Figure 3: The top phyla in the soil, rhizosphere, and root endophytic compartment. (a)** The relative abundances of the top three bacterial phyla are shown for the (left panel) soil, (middle) rhizosphere, and (right) root endophytic compartments. **(b)** The relative abundances of the top two fungal phyla are also shown (as in panel a).

**Supplementary Figure 4: The distribution of bacteria and fungi across samples.** The top (a) bacteria and (b) fungi within the microbiome, as shown in Fig. 2 b, d. In this figure, the taxonomic assignments are included along the right hand side of each heatmap.


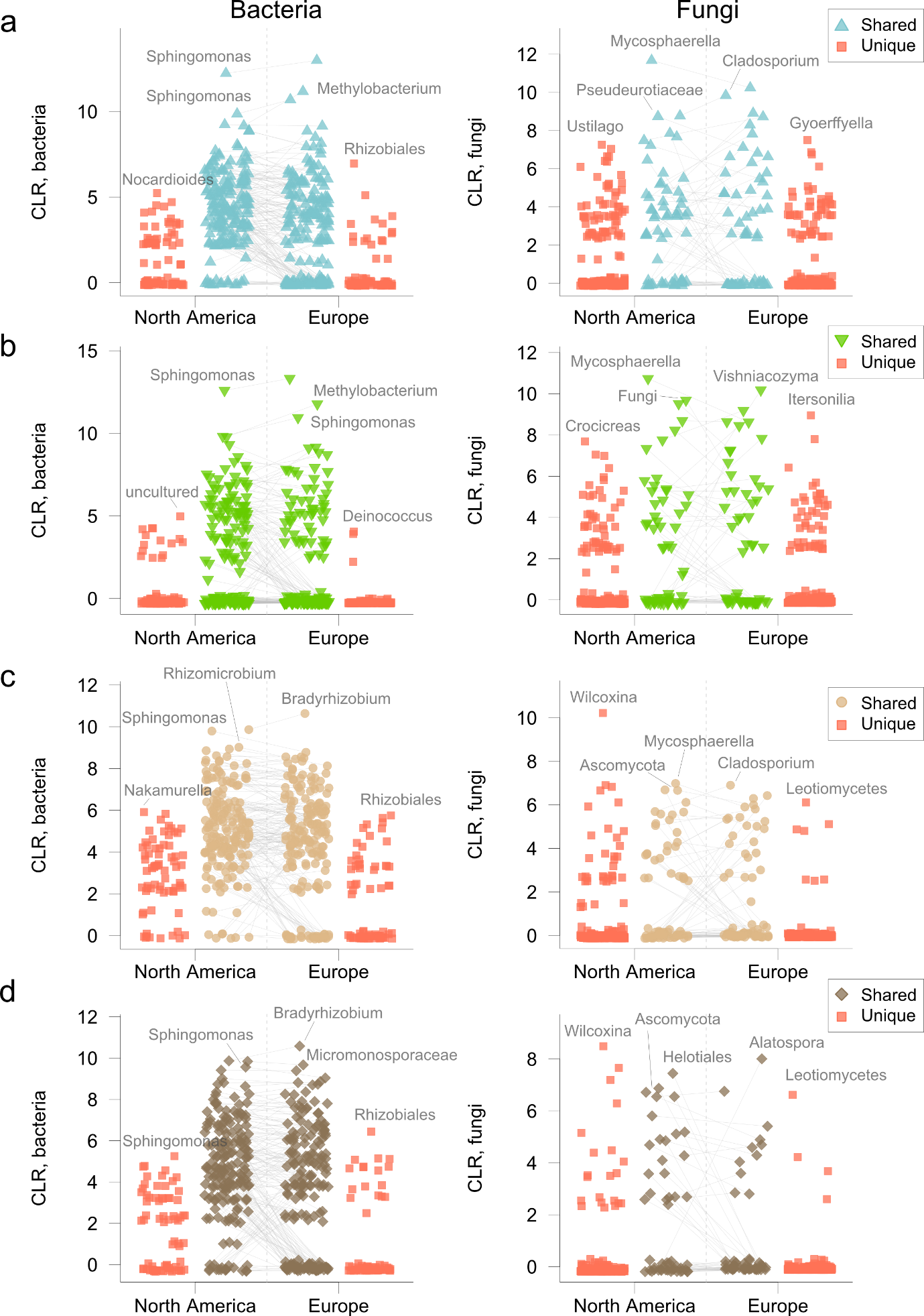


**Supplementary Figure 5: The distribution of bacteria and fungi across transects.** The overlap among the top 250 bacterial and fungal **(a)** leaf epiphytes, **(b)** leaf endophytes, **(c)** root epiphytes, and **(d)** root endophytes, after CLR-normalization. The taxa that are shared across transects are connected with lines. The bacteria are shown on the left side of the figure, while the fungi are shown on the right. The top 5 most abundant taxa are labeled.

**Supplementary Figure 6:** **The pattern of environmental variability across sites.** **(a)** The centered and scaled climatic, geographic, and soil conditions (in rows) at each sampling location (in columns). Missing data are plotted in white. The environmental data are also plotted for the separate **(b)** North American and **(c)** European transects. The Spearman correlation coefficients are shown for **(d)** North American and **(e)** European populations.

**Supplementary Figure 7: The environmental variables that best predict the overall similarity of the plant microbiome.** The results from PCA of North American (NAm, **a, c)** and European samples **(b, d)** are shown for leaf- **(a, b)** and root-associated **(c, d)** communities. For each panel, the fungal communities are plotted in the upper row; the bacterial samples are shown in the bottom row. Epiphytic samples are plotted on the left of each panel, while the endophytes are shown on the right. Each PCA was performed with the top 250 taxa within each community, after center log ratio transformation. Green dashed lines indicate the environmental variables identified using forward selection (FDR *q* ≤ 0.10); the solid lines highlight the top 3 taxa along each axis. *R^2^* values were set to 0 if none of the forward-selected variables passed the *FDR* threshold.


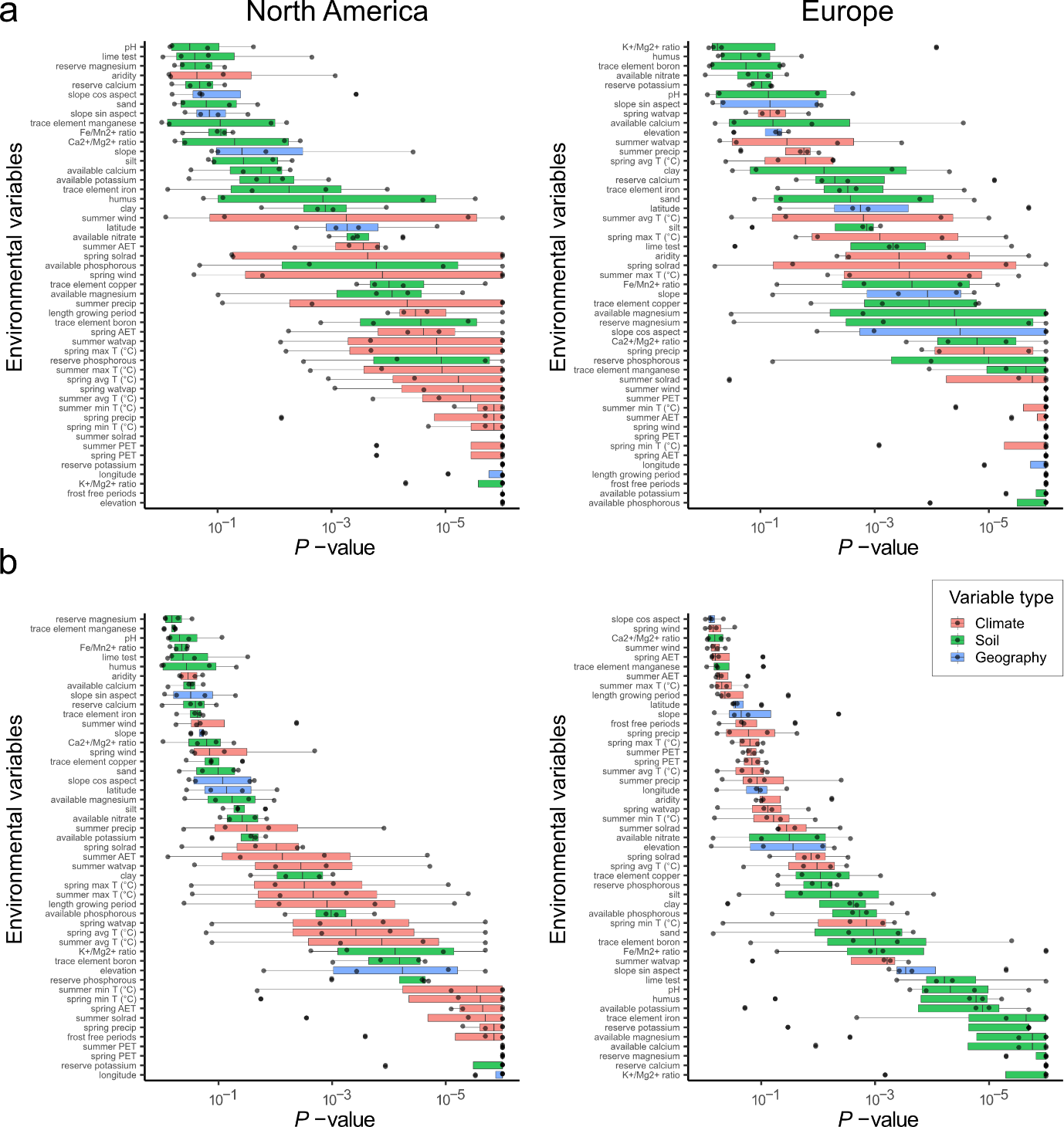


**Supplementary Figure 8: The relationship between the environment and microbiome similarity.** The relationship between the environment and microbiome similarity for fungi **(a)** and bacteria **(b)**. The results from the North American transect are shown on the left; the results from Europe are on the right. The function *envfit*, which is available in the *R* package *vegan*, was used to examine the relationship between the environment and beta diversity. The *P* – values were estimated using 999,999 permutations (minimum possible *P*: 1 × 10^-6^).


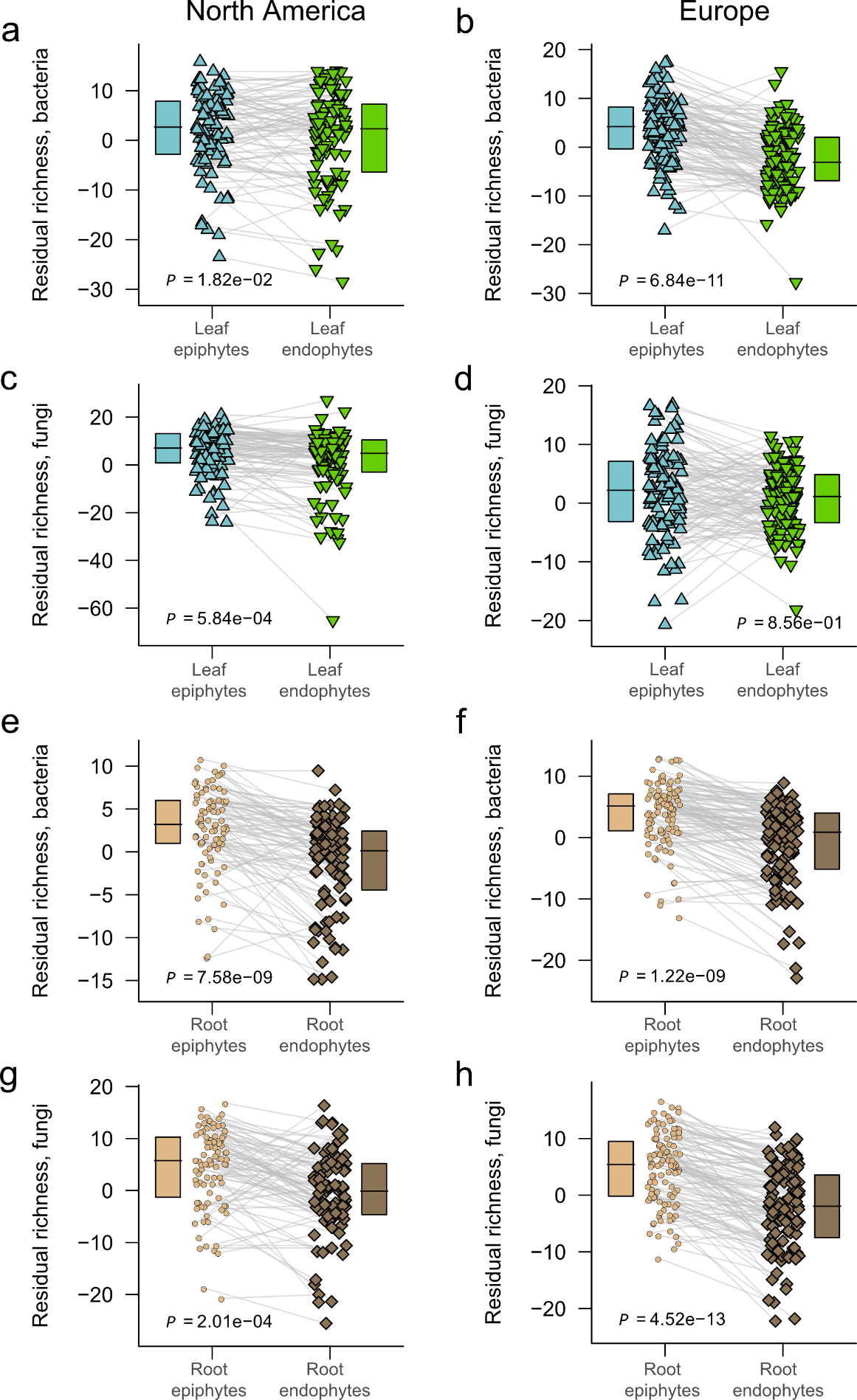


**Supplementary Figure 9: Diversity in the wild strawberry microbiome.** The richness of bacterial **(a-b, e-f)** and fungal **(c-d, g-h)** communities in the leaves of North American **(a, c, e, g)** and European **(b, d, f, h)** samples. Epiphytic samples are plotted to the left side of each panel, while endophytes are on the right. The residuals from quasi-Poisson generalized linear models, which correct for differences in the number of reads among samples, are plotted.

**Supplementary Figure 10: The environmental variables associated with plant-fungal and plant-bacterial richness.** The environmental variables that predict richness in fungal **(a)** and bacterial **(b)** communities. Samples collected along the NAm transect are plotted on the left, and samples collected along the European transect are on the right. To compare the environmental variables that shape the microbial community, compare panels within columns. Quasi-Poisson generalized linear models that control for the number of reads per sample were used. The environmental variables are sorted by (decreasing) *P* – value.

**Supplementary Figure 11: Environment × Environment (E × E) interactions associated with the microbiome.** **(a)** Variation in soil pH for North American and European field sites. **(b)** Variation in calcium availability for North American and European field sites. **(c)** Variation in potassium availability for North American and European field sites. **(d)** The *P* – value matrix from quasi-Poisson generalized linear models investigating E × E interactions within North American samples. **(e)** The matrix of *P* – values for E × E interactions identified within European samples. **(f)** The results from leave-one-out Mantel tests of environmental variables, using the matrices shown in panels (d) and (e). The variable most responsible for the similarity of E × E interactions across transects is the presence of lime (soil carbonate). The *P* – value matrices shown in panels (d) and (e) are -log_10_ transformed.

**Supplementary Tables**

**Supplementary Table 1: The locations of the field sites.**

| SiteID | Site name | Country | Altitude (m) | Latitude | Longitude | Sampling Date |
| --- | --- | --- | --- | --- | --- | --- |
| DUR | Nassenboden | CH | 740 | 47.3359 | 7.5422 | 12.06.17 |
| THAL | Thal | CH | 1110 | 47.2877 | 7.6630 | 12.06.17 |
| RIE | Rieden | CH | 1090 | 47.2231 | 9.0916 | 13.06.17 |
| VAL | Valendas | CH | 1180 | 46.7860 | 9.3120 | 13.06.17 |
| GTR | Gantrisch | CH | 1130 | 46.7475 | 7.3453 | 14.06.17 |
| PER | Pertisau | AUS | 1030 | 47.4290 | 11.6842 | 16.06.17 |
| LHE | Lehen | AUS | 1040 | 46.7510 | 12.5310 | 16.06.17 |
| GLU | Glurns | IT | 1230 | 46.6404 | 10.5549 | 19.06.17 |
| AMB | Altmühlibach | CH | 1000 | 47.0004 | 8.0049 | 20.06.17 |
| SCG | St. Cergue | CH | 1010 | 46.4393 | 6.1609 | 21.06.17 |
| SOZ | Semnoz | F | 985 | 45.8363 | 6.1013 | 21.06.17 |
| VIL | Villarlurin | F | 896 | 45.4564 | 6.5306 | 21.06.17 |
| FAS | Feld am See | AUS | 950 | 46.7528 | 13.7552 | 17.06.17 |
| VRD | Verditz | AUS | 1000 | 46.7038 | 13.8047 | 17.06.17 |
| SLF | Silver Lake Forest | US | 2690 | 40.6089 | -111.5934 | 01.08.17 |
| MIL | Mill D | US | 2278 | 40.6521 | -111.6482 | 01.08.17 |
| WP | White Pines | US | 2347 | 40.5743 | -111.6802 | 02.08.17 |
| UINBC | Uinta Mountains Beaver Creek | US | 2175 | 40.6212 | -111.1458 | 03.08.17 |
| SOP | Soapstone | US | 2543 | 40.5661 | -111.0403 | 03.08.17 |
| TIB | Tibble Fork Reservoir | US | 1981 | 40.4845 | -111.6380 | 04.08.17 |
| SILV | Silver Lake Flat Reservoir | US | 2290 | 40.5068 | -111.6578 | 04.08.17 |
| CASC | Cascade Falls | US | 2263 | 40.4338 | -111.6312 | 04.08.17 |
| CRM | Christmas Meadows | US | 2719 | 40.8476 | -110.8186 | 05.08.17 |
| UINCG | Uinta Campground | US | 2763 | 40.7995 | -110.8752 | 05.08.17 |
| LAM | Lamb's Canyon | US | 2199 | 40.7164 | -111.6224 | 06.08.17 |
| ECY | East Canyon | US | 2217 | 40.8321 | -111.6544 | 06.08.17 |

**Supplementary Table 2. The environmental variables used in analyses.**

| Type | Label | Units | Source | Description |
| --- | --- | --- | --- | --- |
| geography | latitude | degree | GPS | Latitude |
| geography | longitude | degree | GPS | Longitude |
| climate | aridity | mm/  day | See ref.^1^ | This dataset represents the average yearly precipitation divided by average yearly potential evapotranspiration, an aridity index defined by the United Nations Environmental Programme (UNEP). For easier interpretation, aridity was converted into -1 × the raw value of aridity. |
| climate | frost free periods | number of days | See ref.^1^ | Yearly average of the period of days during a year when no frost occurred, or the average of the extreme minimum temperature is higher than 2°C. |
| climate | length growing period | number of days | See ref.^1^ | Yearly average of the days during a year when precipitation exceeds half of the potential evapotranspiration, A period required to evapotranspire an assumed 100mm of water from excess precipitation stored in the soil profile is sometimes added. No provision is made for stored soil moisture. |
| geography | slope | degrees slope | See ref.^2^ | The slope describes the steepness of a given location. |
| climate | spring AET | mm | See ref.^3^ | Monthly average of the actual evapotranspiration in spring, which is defined as the actual rate of water uptake by the plant, which is determined by the level of available water in the soil and combines simultaneously both evaporative losses from the soil surface and transpiration from the plant surface. |
| climate | spring avg T (°C) | °C | See ref.^4^ | Spring average temperature. |
| climate | spring max T (°C) | °C | See ref.^4^ | Spring maximum temperature. |
| climate | spring min T (°C) | °C | See ref.^4^ | Spring minimum temperature. |
| climate | spring PET | mm | See ref.^3^ | Monthly average of the potential evapotranspiration in spring, which is defined as the maximum quantity of water capable of being lost (as water vapor) in a given climate, by a continuous stretch of vegetation covering the whole ground and well supplied with water. |
| climate | spring precip | mm | See ref.^4^ | Spring precipitation. |
| climate | spring solrad | kJ m^-2^day^-1^ | See ref.^4^ | Spring solar radiation. |
| climate | spring water vapor (VP) | kPa | See ref.^4^ | Spring water vapor pressure. |
| climate | spring wind | m/s | See ref.^4^ | Spring wind speed. |
| climate | summer AET | mm | See ref.^3^ | Monthly average of the actual evapotranspiration in summer, which is defined as the actual rate of water uptake by the plant, which is determined by the level of available water in the soil and combines simultaneously both evaporative losses from the soil surface and transpiration from the plant surface. |
| climate | summer avg T (°C) | °C | See ref.^4^ | Summer average temperature. |
| climate | summer max T (°C) | °C | See ref.^4^ | Summer maximum temperature. |
| climate | summer min T (°C) | °C | See ref.^4^ | Summer minimum temperature. |
| climate | summer PET | mm | See ref.^3^ | Monthly average of the potential evapotranspiration in summer, which is defined as the maximum quantity of water capable of being lost (as water vapor) in a given climate, by a continuous stretch of vegetation covering the whole ground and well supplied with water. |
| climate | summer precip | Mm | See ref.^4^ | Summer precipitation. |
| climate | summer solrad | kJ m^-2^day^-1^ | See ref.^4^ | Summer solar radiation. |
| climate | summer water vapor (VP) | kPa | See ref.^4^ | Summer water vapor pressure. |
| climate | summer wind | m s^-1^ | See ref.^4^ | Summer wind speed. |
| soil | clay | percent | LBU* |  |
| soil | silt | percent | LBU* |  |
| soil | lime test | Yes/no | LBU* | The presence of lime |
| soil | humus | percent | LBU* |  |
| soil | sand | percent | LBU* |  |
| soil | pH | pH | LBU* |  |
| soil | available nitrate | mg/kg | LBU* | available NO3 |
| soil | available phosphorous | mg/kg | LBU* | available P |
| soil | available potassium | mg/kg | LBU* | available K |
| soil | available calcium | mg/kg | LBU* | available Ca |
| soil | available magnesium | mg/kg | LBU* | available Mg |
| soil | reserve phosphorous | mg/kg | LBU* | reserve P |
| soil | reserve potassium | mg/kg | LBU* | reserve K |
| soil | reserve calcium | mg/kg | LBU* | reserve Ca |
| soil | reserve magnesium | mg/kg | LBU* | reserve Mg |
| soil | trace element manganese | mg/kg | LBU* | Mn |
| soil | trace element boron | mg/kg | LBU* | B |
| soil | trace element copper | mg/kg | LBU* | Cu |
| soil | trace element iron | mg/kg | LBU* | Fe |
| geography | slope × cos aspect | - | See ref.^2^ | Northing, multiplied by slope. |
| geography | slope × sin aspect | - | See ref.^2^ | Easting, multiplied by slope. |
| geography | elevation | m | GPS | Meters above sea level. |
| soil | K/Mg ratio | - | LBU* | Ratio of available K/Mg |
| soil | Ca/Mg ratio | - | LBU* | Ratio of available Ca/Mg |
| soil | Fe/Mn ratio | - | LBU* | Ratio of available Fe/Mn |

*LBU Labor für Boden- und Umweltanalytik, Eric Schweizer AG, Postfach 150, CH-3602 Thun, Switzerland: http://lbu.ch

**Supplementary Table 3: The relationship between plant microbiome richness and E × E interactions.** The top E × E interactions associated with combined bacterial and fungal richness in the microbiome of plants collected in North America (NAm) and Europe. The *P* – values are from quasi-Poisson generalized linear models in which, to control for differences in the number of reads among samples, the log of the number of reads in each sample was included as an offset.

| Rank | Transect | Organ | Habitat | Environmental variables | *P* - value |
| --- | --- | --- | --- | --- | --- |
| 1 | Europe | Leaf | epiphytes | available_magnesium × pH | 8.04E-10 |
| 2 | Europe | Leaf | epiphytes | available_calcium × pH | 9.23E-10 |
| 3 | Europe | Leaf | epiphytes | pH × summer_wind | 2.65E-09 |
| *1* | *Europe* | *Leaf* | *endophytes* | *lime_test × slope* | *0.003140042* |
| *2* | *Europe* | *Leaf* | *endophytes* | *summer_solrad × summer_wind* | *0.003703537* |
| *3* | *Europe* | *Leaf* | *endophytes* | *longitude × summer_wind* | *0.004084893* |
| 1 | Europe | Root | epiphytes | available_calcium × spring_watvap | 9.57E-14 |
| 2 | Europe | Root | epiphytes | available_potassium × lime_test | 9.06E-13 |
| 3 | Europe | Root | epiphytes | available_calcium × pH | 1.21E-12 |
| *1* | *Europe* | *Root* | *endophytes* | *longitude × spring_watvap* | *8.05E-10* |
| *2* | *Europe* | *Root* | *endophytes* | *available_calcium × pH* | *2.12E-09* |
| *3* | *Europe* | *Root* | *endophytes* | *fe_mn_ratio × longitude* | *2.52E-09* |
| 1 | NAm | Leaf | epiphytes | longitude ×  trace_element_copper | 8.67E-16 |
| 2 | NAm | Leaf | epiphytes | latitude × trace_element_copper | 1.58E-14 |
| 3 | NAm | Leaf | epiphytes | available_nitrate × trace_element_copper | 2.59E-14 |
| *1* | *NAm* | *Leaf* | *endophytes* | *available_nitrate × trace_element_copper* | *6.83E-11* |
| *2* | *NAm* | *Leaf* | *endophytes* | *reserve_magnesium × summer_precip* | *2.14E-09* |
| *3* | *NAm* | *Leaf* | *endophytes* | *slope × summer_wind* | *9.53E-09* |
| 1 | NAm | Root | epiphytes | available_nitrate × trace_element_copper | 1.58E-12 |
| 2 | NAm | Root | epiphytes | reserve_magnesium × summer_precip | 2.78E-11 |
| 3 | NAm | Root | epiphytes | available_magnesium × lime_test | 4.44E-11 |
| *1* | *NAm* | *Root* | *endophytes* | *reserve_magnesium × spring_wind* | *3.68E-06* |
| *2* | *NAm* | *Root* | *endophytes* | *reserve_magnesium × summer_precip* | *5.28E-06* |
| *3* | *NAm* | *Root* | *endophytes* | *latitude × lime_test* | *7.07E-06* |

**Supplementary Data file information**

**Supplementary Data 1: Sample metadata.** The description of each Amplicon library.

**Supplementary Data 2. The raw environmental data.** The environmental variables are further described in Supplementary Table 2.

**Supplementary Data 3. The taxa enriched in the wild strawberry microbiome. (**Sheet 1) Differences in the taxonomy of the leaf and root microbiome. (Sheet 2) Differences among soil and root-epiphytic (rhizosphere) bacteria. (Sheet 3) The differences among soil and root-epiphytic (rhizosphere) fungi. The North American transect is abbreviated NAm. Each enrichment analysis was performed with quasi-Poisson generalized linear models (GLMs).

**Supplementary References**

1. FAO GeoNetwork. http://www.fao.org/geonetwork/.

2. USGS digital elevation dataset SRTM mission. https://www.usgs.gov/centers/eros/science/usgs-eros-archive-digital-elevation-shuttle-radar-topography-mission-srtm-1-arc?qt-science_center_objects=0#qt-science_center_objects (2020).

3. Global GIS : Global Climate Database : Actual evapotranspiration in EarthWorks. https://earthworks.stanford.edu/catalog/harvard-glb-claet.

4. Fick, S. E. & Hijmans, R. J. WorldClim 2: new 1-km spatial resolution climate surfaces for global land areas. *Int. J. Climatol.* **37**, 4302–4315 (2017).
